# p53 as a Dichotomous Regulator of Liver Disease: The Dose Makes the Medicine

**DOI:** 10.3390/ijms19030921

**Published:** 2018-03-20

**Authors:** Jelena Krstic, Markus Galhuber, Tim J. Schulz, Michael Schupp, Andreas Prokesch

**Affiliations:** 1 Gottfried Schatz Research Center for Cell Signaling, Metabolism & Aging, Medical University of Graz, 8010 Graz, Austria; jelena.krstic@medunigraz.at (J.K.); markus.galhuber@medunigraz.at (M.G.); 2 Department of Adipocyte Development and Nutrition, German Institute of Human Nutrition, Potsdam-Rehhbrücke, 14558 Nuthetal, Germany; tim.schulz@dife.de; 3 German Center for Diabetes Research (DZD), 85764 München-Neuherberg, Germany; 4 Institute of Nutritional Science, University of Potsdam, 14558 Nuthetal, Germany; 5 Charité—Universitätsmedizin Berlin, Corporate Member of Freie Universität Berlin, Humboldt-Universität zu Berlin, and Berlin Institute of Health, Institute of Pharmacology, Center for Cardiovascular Research, 10117 Berlin, Germany; michael.schupp@charite.de; 6 BioTechMed-Graz, 8010 Graz, Austria

**Keywords:** p53, liver disease, insulin resistance, non-alcoholic fatty liver disease, non-alcoholic steatohepatitis, hepatocellular carcinoma, liver regeneration, mouse models

## Abstract

Lifestyle-related disorders, such as the metabolic syndrome, have become a primary risk factor for the development of liver pathologies that can progress from hepatic steatosis, hepatic insulin resistance, steatohepatitis, fibrosis and cirrhosis, to the most severe condition of hepatocellular carcinoma (HCC). While the prevalence of liver pathologies is steadily increasing in modern societies, there are currently no approved drugs other than chemotherapeutic intervention in late stage HCC. Hence, there is a pressing need to identify and investigate causative molecular pathways that can yield new therapeutic avenues. The transcription factor p53 is well established as a tumor suppressor and has recently been described as a central metabolic player both in physiological and pathological settings. Given that liver is a dynamic tissue with direct exposition to ingested nutrients, hepatic p53, by integrating cellular stress response, metabolism and cell cycle regulation, has emerged as an important regulator of liver homeostasis and dysfunction. The underlying evidence is reviewed herein, with a focus on clinical data and animal studies that highlight a direct influence of p53 activity on different stages of liver diseases. Based on current literature showing that activation of p53 signaling can either attenuate or fuel liver disease, we herein discuss the hypothesis that, while hyper-activation or loss of function can cause disease, moderate induction of hepatic p53 within physiological margins could be beneficial in the prevention and treatment of liver pathologies. Hence, stimuli that lead to a moderate and temporary p53 activation could present new therapeutic approaches through several entry points in the cascade from hepatic steatosis to HCC.

## 1. Liver Disease in the Age of Metabolic Syndrome

The term “liver disease’ encompasses a spectrum of pathologies that includes, but is not limited to, hepatosteatosis, hepatic insulin resistance, steatohepatitis, liver fibrosis and cirrhosis and hepatocellular carcinoma (HCC) [1]. While failure of liver function can occur at several stages, HCC is often regarded as the end stage of the progression of alcoholic liver disease (ALD) or non-alcoholic fatty liver disease (NAFLD), with varying incidences [2]. Whereas some models suggest a defined, successive cascade in the etiology of liver disease (steatosis and hepatic insulin resistance leading to steatohepatitis, which may lead to fibrosis/cirrhosis, and ultimately to HCC), other reports paint a less clear picture. For example, a number of mouse models that develop fatty liver disease are not insulin resistant [3], and some people with NAFLD develop fibrosis without preceding inflammatory conditions typical for non-alcoholic steatohepatitis (NASH) [4]. Furthermore, HCC in patients has been reported without any signs of fibrosis/cirrhosis [5].

Besides genetic [6] and epigenetic (mostly differential DNA methylation [7]) aberrations, infection with hepatitis B and C virus and aflatoxin B1 exposure were historically prevalent risk factors for liver disease development. Prevention of aflatoxin B1 ingestion and vaccines against hepatitis viruses have massively curtailed the epidemiologic impact of those disease-causing agents [8]. However, in modern societies, these traditional risk factors have been replaced by lifestyle-related diseases caused by chronic alcohol and/or caloric over-consumption [9,10]. In particular, malnutrition through chronic caloric overload can precipitate in the development of metabolic syndrome, a cluster of pathologies, including adiposity, dyslipidemia, insulin resistance and hypertension, that together culminate in the increased risk of type 2 diabetes mellitus and cardiovascular complications [11]. There is an increasingly strong association between liver disease and metabolic syndrome [1]. This connection between liver disease and metabolic syndrome through malnutrition seems plausible given the liver’s immediate exposure to digested nutrients that enter the splanchnic circulation and are directly relayed to hepatic sinusoids via the portal vein. For instance, the average per-person fructose consumption has been surging due to a preference for readily-available high-fructose corn syrup by the food industry [12]. Unlike glucose, fructose is almost entirely taken up by the liver and serves as a carbon source for de novo lipogenesis, thereby fueling the development of steatosis and NAFLD [12]. Considering the global rise in the prevalence of obesity and the concurrent surge in NAFLD (10–40% of adults [1]), it is imperative to improve diagnostic tools and pharmacological interventions to deal with the increasing burden of liver disease. In terms of diagnostics, liver biopsy, which poses a health risk, is still the best method for the detection of hepatic pathologies, while the search for biomarkers is ongoing, and non-invasive imaging methods (e.g., magnetic resonance) are being improved (for details see [13]). Weight loss through a combination of a healthy diet and exercise is the first line of treatment for NAFLD, and drugs treating other components of the metabolic syndrome are being used as adjunct therapies [14]. However, although there are currently more than 200 clinical trials for NAFLD treatments [8], there is no approved drug specifically targeting any stage of liver disease, with the exception of the chemotherapeutic sorafenib in late stage HCC [15]. This multi-kinase inhibitor results in a small survival benefit of only three months, and patients often develop resistance [2]. Hence, there is a pressing need to investigate the molecular pathways involved in the distinct stages of liver disease and to translate this knowledge into novel therapeutic possibilities.

p53 is a well-described tumor suppressor that regulates DNA repair and cell cycle arrest under oncogenic stress and, in cases of prolonged stress exposure, apoptosis [16,17]. The importance of p53 in controlling tumorigenesis becomes evident in patients who inherit mutant alleles, a condition known as Li-Fraumeni syndrome, which results in an extraordinary high, early-onset cancer risk [18]. While cytoplasmic actions of p53 have been reported [19], it mainly functions as a transcription factor. This notion is supported by the fact that most TP53 missense mutations (i.e., hot-spot mutations) occur in the DNA-binding region [20]. Meta-analyses from genome-wide datasets revealed that the set of target genes regulated by p53 activation is largely specific to the cell type (i.e., context-dependent), differentiation state and the nature and degree of the activating stimulus, with only a small set of common targets over all investigated conditions [21]. More recently, p53 has been recognized as a bona fide regulator of different metabolic pathways [22,23], a function that also seems to be highly context-dependent. For instance, p53 acts to limit glycolysis in breast cancer cells, while it induces glycolytic enzymes in muscle cells [24]. Hence, the role of p53 needs to be investigated and described in defined cellular and tissue contexts, because single findings about p53-dependent effects can hardly be generalized. Here we aim to review the p53-relevant literature in relation to liver pathologies. With almost four decades of p53 research and over 88,000 PubMed entries containing “p53” in the title and/or abstract by the end of 2017, it is inherent that p53 reviews are destined to be incomplete, even if they are restricted to a defined subtopic. Therefore, we focus on patient data and in vivo mouse studies that point out an influence of p53 activity on liver diseases and put emphasis on studies that directly implicate p53 via gain- and loss-of-function approaches. Although in vitro studies are instrumental for understanding the molecular mechanisms of p53 in liver cells, reviewing them in detail is beyond the scope of this article. Furthermore, we will limit the discussion on liver pathologies related to the metabolic syndrome and other non-alcoholic causes, thereby largely disregarding alcoholic liver disease, fungal and viral disease mechanisms, which are reviewed elsewhere [25].

## 2. p53 in the Development of Non-Alcoholic Fatty Liver Disease

NAFLD is characterized by hepatosteatosis, an abnormal accretion of triglycerides in more than 5% of hepatocytes, which is considered a common precursor of severe hepatocyte injury and chronic liver disease [1]. With the high incidence of NAFLD in adults (20–50% in Western countries [26,27,28,29]) and children (as high as 80% in obese Chinese children [30]) and limited therapeutic options, there is a need to understand the exact mechanisms underlying NAFLD. A major substrate for triglyceride biosynthesis are intrahepatic fatty acids that can be derived from increased fatty acid influx (from adipose tissue in the case of peripheral insulin-resistance or during fasting), from chylomicron-remnants after dietary lipid ingestion and from carbohydrate-derived hepatic acetyl-CoA fueling de novo lipogenesis [31].

**Figure 1:**
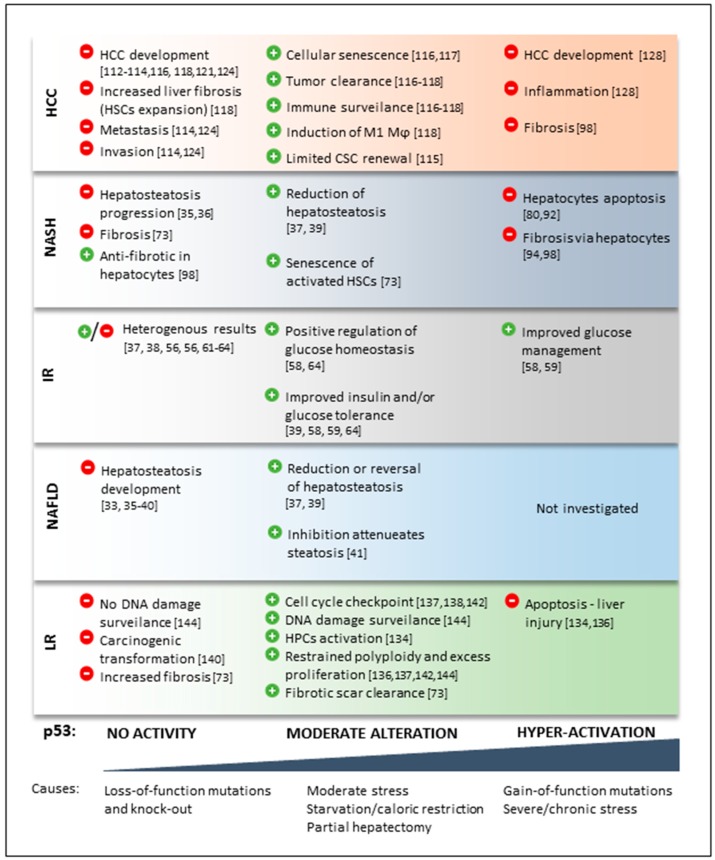
p53 level-dependent outcomes in liver diseases. Various aspects of liver disease are affected by p53 levels in vivo. Please refer to the text for further details. Red circles depict negative, while green circles depict positive outcomes of p53 level alterations. Abbreviations: HCC, hepatocellular carcinoma; NASH, non-alcoholic steatohepatitis; NAFLD, non-alcoholic fatty liver disease; IR, hepatic insulin resistance; LR, liver regeneration; HSCs, hepatic stellate cells; Mϕ, macrophage; CSC, cancer stem cell; HPCs, hepatic progenitor cells.

Owing to the limited availability of liver biopsies, data on p53 in NAFLD patients are scarce. An immunohistochemical study in 84 patients reported a slightly positive correlation of p53 expression and severity of liver disease [32]. In mice, p53 was first implicated in hepatic steatosis in an observational study in 2004, where it was shown to be induced in nuclei of hepatocytes from two mouse models with fatty liver disease [33]. Functional insights arose from the first p53 knock-out mice, which die around six months of age due to the development of severe lymphomas or sarcomas [34]. However, young p53 knock-out mice can be helpful in determining p53 functions in non-cancerous tissues. Although this p53-deficient mouse strain was originally described in 1992 [34], its liver phenotype was not reported until about 20 years later [35,36]. Wang et al. showed that young p53 knock-out mice develop hepatic steatosis, which was even more pronounced during a high-fat diet (HFD). The proposed mechanism involved the enzyme aromatase, a direct transcriptional target of p53, which is responsible for the conversion from androgens into estrogen [36]. Consequently, in HFD-fed p53-deficient mice, lower aromatase expression resulted in a higher serum testosterone/estradiol ratio, leading to triglyceride accumulation. This hepatic steatosis was completely reversed by transgenic overexpression of aromatase in the p53 knock-out background [36]. Another study also showed increased steatosis in young p53 knock-out mice accompanied by higher serum alanine transaminase and aspartate transaminase levels, when compared to wild-type mice [37]. This phenotype was exacerbated by feeding mice a HFD and could be reversed by re-expression of adenoviral p53. The underlying mechanism described in this work involved a compensatory upregulation of TAp63 as a consequence of p53 loss, with subsequent induction of I-kappa-B-kinase beta (IKKβ) and endoplasmic reticulum stress [37]. Interestingly, hepatic p53 knock-out induced in adult p53-floxed mice (using either AAV8 or adenoviral Cre recombinase expression [37,38]) also leads to hepatosteatosis on a regular diet, indicating that acute deletion of p53 in the liver of adult mice is sufficiently driving lipid accumulation by mechanisms independent of potential developmental or adaptive effects that may occur in long-term knock-out models. Furthermore, activation of p53 signaling with long-term, low-dose doxorubicin treatment reduced liver triglyceride content in diet-induced NAFLD mouse models [39]. Supporting these findings, mouse double minute 2 homolog (Mdm2) transgenic mice with an amino acid change at codon 305 (C305F), a strain with reduced hepatic p53 signaling due to decreased sequestration of Mdm2 to ribosomal proteins under nutrient stress, display severe hepatosteatosis in the fasted state via modulation of fatty acid oxidation by the p53 target malonyl-CoA decarboxylase [40]. In contrast to the above findings that show that p53 loss evokes liver lipid accumulation, a pharmacological approach using the p53 inhibitor pifithrin-α showed attenuation of HFD-induced steatosis [41]. However, pifithrin-α treatment prevented weight gain without decreasing food intake under HFD, which could be the primary determinant for decreased hepatosteatosis in this model. Moreover, pifithrin-α was shown to exert p53-independent effects that could be responsible for the discrepancies between genetic models and this pharmacological approach to reduce p53 signaling [42,43]. Consistent with the notion that p53 is important for the control of liver lipid homeostasis, upregulation of p53 mRNA by anti-sense oligo-mediated quenching of its upstream inhibitor miR-21 led to a reduction in HFD-induced steatosis in mice [44]. Other known p53-regulated pathways related to lipid metabolism (reviewed elsewhere [22,45,46,47]) are mitochondrial respiration (e.g., cytochrome C oxidase assembly protein (SCO2)) and fatty acid oxidation (e.g., carnitine palmitoyltransferase (CTP1C), lipin 1 (LPIN1)), the pentose phosphate/NADPH pathway (e.g., glucose-6-phosphate dehydrogenase (G6PD)), glycolysis (e.g., TP53-induced glycolysis regulatory phosphatase (TIGAR), glucose transporters (GLUT1/4)) or the mevalonate pathway (via sterol regulatory element binding transcription factor 1 (SREBP1c)). However, p53 cross-talk with these pathways has been demonstrated in cell systems other than hepatocytes and has yet to be investigated in the context of NAFLD. In summary, there is a surplus of evidence portraying p53 as a key regulator of hepatocyte lipid metabolism, where reduction in p53 activity elicits and activation attenuates hepatosteatosis (Figure 1).

## 3. p53 in the Regulation of Hepatic Insulin Resistance

Hepatic insulin resistance is epidemiologically correlated with steatosis [1]. Several mechanisms linked to impairment of hepatic insulin receptor signaling have been described, including diacylglycerol-mediated protein kinase C epsilon type (PKCε) activation [47], as well as Akt serine/threonine kinase-dependent and SREBP1-dependent pathways [48,49]. Additionally, extra-hepatic driver mechanisms of insulin resistance-mediated steatosis have been suggested, such as impairment of insulin-mediated suppression of lipolysis in white adipose tissue or insulin resistance in skeletal muscle [50]. Data from lipodystrophic mice and patients with generalized lipodystrophy show a co-occurrence of hepatic insulin resistance and steatosis as a consequence of ectopic lipid deposition in the absence of white adipose tissue [51,52]. However, several transgenic and knock-out mouse models that develop fatty liver show normal glucose management and insulin sensitivity, arguing for a dissociation of fatty liver disease and hepatic insulin resistance (and therefore, the development of type 2 diabetes mellitus, summarized in [3]). Hence, we chose to discuss the influence of p53 on steatosis and hepatic insulin sensitivity in separate sections.

The gold standard for measuring hepatic insulin resistance is to determine hepatic glucose output during a hyperinsulinemic-euglycemic clamp in combination with isotope-labeled tracer studies [53,54]. Because this method is very difficult to perform, most studies use insulin tolerance tests (ITT) and/or glucose tolerance tests (GTT) in p53-relevant mouse models as a proxy for assessing systemic insulin tolerance [54].

Knock-in mice with a mutation of serine 18 (corresponding to human serine 15) to alanine, a residue that is phosphorylated by different upstream kinases (e.g., ATM, AMPK) resulting in p53 protein stabilization [55], were characterized comprehensively in terms of glucose homeostasis. These p53 (S18A) mice, despite p53 destabilization, remained tumor-free throughout the study and showed impaired glucose and insulin tolerance. Importantly, hepatic glucose production under hyperinsulinemic clamp conditions was not suppressed, which is a clear indicator of hepatic insulin resistance [56]. Moreover, glucose management was shown to be dependent on the dosage of the N-terminal transactivation domain of p53, and mice expressing extra p53 alleles (“super-p53” mice [57]) showed improved glucose tolerance [58]. Similar to these findings in “super-p53” mice, another report showed a reduction of fasting blood glucose and an improvement of glucose tolerance after hepatic overexpression of p53 in a diabetic mouse model [59]. In another study, different doses of doxorubicin were intraperitoneally or orally applied to HFD-fed mice to activate p53. This led to a dose-dependent improvement of glucose tolerance [39]. In contrast to the above studies indicating that wild-type p53 is necessary for, and its activation enhances, (hepatic) insulin sensitivity, an increase of hepatic p53 expression in rat models of alcohol-induced liver disease was associated with insulin resistance [60]. However, this study in rats reported an impairment of glucose metabolism in three different rat strains (Fisher, Sprague-Dawley, Long-Evans), while p53 and its target TIGAR were only induced in Long-Evans rats [60]. Furthermore, due to the lack of a p53-deficient system in this study, it cannot be deduced if p53 activation in Long-Evans rats is the cause or consequence of ethanol-mediated liver disease. Taken together, most gain-of-function studies suggest p53 as a positive regulator of glucose management with improvement of (hepatic) insulin sensitivity upon activation of p53 signaling.

The interpretation of the available data from various loss-of-function models is less clear due to heterogeneous results. For instance, several studies performing a pyruvate tolerance test report either an improvement [61], no change [62] or an impairment [63,64] in gluconeogenic ability in whole body p53 knock-out mice compared to wild-type mice. These discrepancies might be due to differences in experimental protocols, dietary composition or age of p53 knock-out mice. In agreement with the studies showing impaired glucose homeostasis, our own data in adeno-Cre-treated p53-floxed mice showed fasting hypoglycemia and a concomitant accumulation of gluconeogenic amino acids under starvation, altogether suggesting an impairment of glucose homeostasis in absence of hepatic p53 under starvation [38]. In another study using young whole body p53 knock-out mice fed an HFD, the authors reported a slight improvement of insulin tolerance with no change in glucose tolerance in male mice, while in female mice, glucose tolerance was improved and insulin tolerance unchanged [37]. The reasons for these sex-specific differences in glucose metabolism in germ-line p53 knock-out mice are unknown. In the same study, knocking out hepatic p53 by AAV8-Cre injection into p53-floxed mice under chow diet left insulin and glucose tolerance unchanged despite an increase in hepatic triglyceride levels [37]. Hence, due to experimental differences, loss-of-function models have not always supported a role of p53 as an attenuating factor in hepatic insulin resistance, as largely suggested by data from p53 activation studies.

An intriguing study compared glucose metabolism in two transgenic mouse lines harboring either a variant with a proline (P72) or an arginine (R72) on position 72 of the human p53 protein [64]. These variants represent two major human populations with a higher frequency of R72 in people living in higher geographic latitude, while the P72 is the equatorial variant that represents the ancestral version also found in lower mammals [65]. Interestingly, R72 mice showed significantly more adiposity when fed a HFD, compared to P72 mice. In agreement with a strong association of the R72 variant with diabetes in human populations [66], measurements from ITT, GTT and hyperinsulinemic-euglycemic clamp indicated increased hepatic insulin resistance in HFD-fed R72 mice compared to P72 mice [64]. This study, together with the data from p53(S18A) mice, underlines the importance of the p53 status in the development of metabolic pathologies such as hepatic insulin resistance and suggests a contribution of p53 mutations to the variation in NAFLD incidence between ethnicities [67].

Several mechanistic connections between p53 and cellular glucose metabolism have been described (reviewed in [22,46,68]). These include direct protein-protein interaction between p53 and peroxisome proliferator-activated receptor gamma coactivator 1 alpha (Pgc1α) under metabolic stress [63]; the p53-SIRT6-FoxO1 axis in the regulation of gluconeogenesis [61]; p53 interaction with G6PD to restrict substrate flux into the pentose phosphate pathway [35]; and direct transcriptional regulation of genes coding for gluconeogenic enzymes [64,69], glycolytic regulators (e.g., *TIGAR* [70]) and glucose transporters [62]. Furthermore, some studies have implicated p53 in the maintenance of liver glycogen stores [38,59,71], which could have an impact on glucose homeostasis. Despite a fair amount of studies, a clear picture on p53′s role in hepatic insulin resistance has yet to emerge. In particular, more studies investigating the above-mentioned mechanisms in a liver-specific context combined with assays directly probing hepatic insulin sensitivity might resolve some of the existing discrepancies. However, from the data available, it is apparent that hepatic p53 signaling is an important node in the regulation of glucose homeostasis [22] and systemic insulin sensitivity [72], and could therefore be a worthwhile therapeutic target for the treatment of hepatic and/or systemic insulin resistance (Figure 1).

## 4. p53 in the Development of Non-Alcoholic Steatohepatitis

The estimations of NASH prevalence in the population varies between different studies and depends on patient inclusion criteria, diagnostic methods and investigated cohort location or ethnic background. It is estimated that between 6% and 55% of NAFLD patients progress to a phenotype that is characterized by elevated levels of inflammation, also known as NASH [1]. Clinically, the distinction between overt steatohepatitis and the fatty liver phenotype is difficult, but important, as NASH patients are at greater risk of developing fibrosis, cirrhosis and even HCC [1]. A collective body of evidence suggests that different promoting factors can determine the progression from NAFLD to NASH. Disease-progressing factors include excess fatty acids, bacterial endotoxins from the gut or damage-associated molecular patterns (DAMPs) from dying hepatocytes, which trigger inflammatory reactions. Excess hepatic lipid content, for example, exerts lipotoxic effects via increased reactive oxygen species (ROS) and oxidative stress, which consequently fuels lipid peroxidation and upregulation of pro-inflammatory cytokines (interleukin-6 (IL-6), tumor necrosis factor α (TNFα)) and adipokines (adiponectin, leptin). Further disease-promoting links were made to cellular senescence and the activation of hepatic stellate cells (HSCs) [73]. In NASH, a combination of these factors can ultimately lead to hepatocellular ballooning followed by zonal hepatocyte injury and either necrotic cell death or programmed apoptosis. These events can further fuel inflammatory processes and induce a vicious cycle [74]. The promotion to NASH has traditionally been described as a “two-hit-model” according to which a first step, steatosis-inducing lipotoxic effects, is followed by a second stage of chronic inflammatory stress [75], which ultimately leads to advanced liver pathologies like fibrosis, cirrhosis or HCC. The exact numbers of incidences for each disease characteristic remains elusive for the general population (discussed in detail in [76]). However, it is important to note that, opposing the “two-hit-model”, clinical data question the proposed linearity of disease progression. For example, late disease stages like HCC may occur in both cirrhotic and non-cirrhotic NASH patients [76]. Therefore, the contemporary theory of NASH development and progression proposes a more complex model that includes multiple steps where it remains unclear if the promoting events appear sequentially or in parallel order [77]. However, it is established that the predominant inducer of liver fibrosis and cirrhosis encompasses inflammation-mediated activation of HSCs, which, once activated, promote fibrotic scar tissue formation by actively remodeling the extracellular matrix (ECM) in the liver [78].

p53 is well known to regulate several factors of NASH disease progression, such as ROS production, cellular senescence or apoptosis. Apoptosis was suggested to result from lipotoxicity, leading to local injuries and subsequent tissue inflammation [79]. As p53 is increasingly portrayed as key regulator of lipid metabolism [45], its involvement in lipotoxicity-mediated NASH disease progression is plausible. It could be hypothesized that stabilized p53 has beneficial effects on liver hepatocytes by positively regulating β-oxidation [45], thereby keeping fatty acid and triglyceride levels within metabolically healthy limits. Whenever these margins are exceeded, pathologically-elevated p53 levels could induce apoptosis and therefore NASH progression. A recent study of two different diet-induced mouse models of steatohepatitis, HFD and methionine-choline-deficient diet (MCD), found that pharmacological, low-dose doxorubicin activation of p53 ameliorates liver injury, further underlining a possible dose-dependent mode of action of p53 [39]. The low-dose doxorubicin activation of p53 in these models did not affect cell viability or apoptosis. Furthermore, increased fatty acid oxidation, decreased de novo fatty acid synthesis, reduced inflammation and lowered ER stress were found to be correlated with the attenuation of liver injury [39]. This argues for a positive effect of moderate p53 activation on liver lipid metabolism and the prevention of NASH-associated liver pathology.

In contrast to moderate activation, excess levels of p53 could lead to undesired cellular fates, including hepatocyte injury followed by cell death, which in NAFLD are strongly linked to disease progression. To decipher the relevance of intrinsic and extrinsic apoptosis pathways in experimental NASH, one study investigated cytokine receptor expression and p53-mediated initiation of apoptosis in the MCD mouse model of NASH [80]. In this study, hepatic p53 expression was found to be significantly increased after only five days of MCD feeding. This upregulation was retained throughout a time course of eight weeks, indicating a close relationship between nutrition-related stressors and p53 expression in this steatohepatitis model. A concomitant decrease in B-cell lymphoma-extra-large (Bcl-XL) levels and enhanced BH3 interacting domain death agonist (Bid) cleavage to tBid together confirmed an upregulation of mitochondrial, i.e., intrinsic apoptosis, which was not recovered upon nutritional repletion [80]. Interestingly, together with p53 activation, a reciprocal reduction of insulin-like growth factor-1 (IGF-1) was found, which was also linked in other studies to the progression from non-pathogenic steatosis to overt NASH [81,82]. Regarding extrinsic stress mediation, the authors hypothesize that upregulated mRNA expression of the death receptor TRAIL is possibly linked to p53 transactivation [80], as found in earlier studies [83,84]. However, because p53-deficient mouse models were not used in this study, a causal role of p53 cannot be claimed. Despite this, the data suggest that p53 could represent a molecular link between intrinsic and extrinsic pathways in NASH by promoting apoptosis in the setting of hepatic steatosis.

Focusing on intrinsic triggers of cellular damage, p53 mediates stress signaling in a number of ways. Lipotoxicity is clearly a major driver of unfavorable cellular events in NASH and liver steatosis, as already reviewed elsewhere [85]. Beyond the toxic effects of excess lipids, growing evidence substantiates a role of p53-related miRNAs in association with NASH development. Among the many miRNAs involved in hepatic metabolism, miRNA-34a has been investigated in greater detail. Initially found to be directly activated by p53 [86,87] and differentially upregulated in human NASH samples [88,89], it was also shown to be upregulated in the MCD mouse model for NASH [90]. Strikingly, since plasma levels of miRNA-34a were upregulated and associated with disease severity also in NAFLD mouse models, it was suggested as a biomarker for susceptibility to liver injury in fatty liver disease [91]. Establishing another link of miRNA-34a and p53 to liver lipid metabolism, data from Xu et al. suggest that in NASH patients, p53, free fatty acids and cholesterol may act synergistically via different pathways to inactivate the hepatocyte nuclear factor 4α (HNFα) via miRNA-34a, ultimately leading to reduced lipoprotein secretion and subsequent steatosis [89]. These data argue for miRNA-34a as a critical factor in the pathogenesis of fatty liver disease, regulation of plasma lipids and lipoprotein metabolism. Mechanistically, miRNA-34a is located downstream of c-Jun N-terminal Kinase 1 (JNK)/c-Jun and upstream of Sirtuin 1 (silent mating type information regulation 2 homolog, Sirt1), an axis ultimately converging on the p53-dependent pro-apoptotic pathway [92,93]. As Sirt1 has been described as negative regulator of p53 activity via de-acetylation, miRNA-34a stabilizes p53 by downregulating Sirt1. In turn, functioning as a positive regulator, activated p53 further drives miRNA-34a expression and thereby potentially enhances its own activity. Taken together, miR-34a abundance in hepatocytes could amplify p53 activity and may contribute to fine-tuning of p53 in hepatocytes. Bile acids also potentially initiate hepatic stress and may trigger apoptotic pathways also via miRNA-34a activation. For instance, one study in rat liver, which was focusing on the apoptosis-inducing effects of bile acids, reported a deoxycholic acid-induced miRNA-34a expression that also blunted Sirt1-mediated downstream inactivation of p53 signaling [93].

To determine whether the miRNA-34a/SIRT1/p53 signaling pathway is also associated with liver fibrosis in progressive NASH, its activation has been investigated in a carbon tetrachloride (CCl_4_)-induced rat liver fibrosis model. In this fibrosis model, miRNA-34a activation, Sirt1 reduction and p53-acetylation correlated well with apoptosis in hepatocytes, but not HSCs [94]. In another study, in a CCl_4_-induced mouse model, miRNA-34a was associated with liver fibrosis and linked, among others, to metabolic pathways and the p53 signaling pathway [95].

A mediator of p53-dependent apoptosis, p66Shc, was described in the nutritional steatohepatitis model using the MCD diet. In rat hepatocytes, p66Shc functions downstream of p53 and induces ROS accumulation, ultimately leading to activation of apoptosis [96]. In this study, the hepatic protein expression levels of p53 and its downstream target p21 increased significantly in the NASH model compared to animals fed a control diet. Upon pharmacological inhibition by pifithrin-α, or whole-body genetic disruption of p53 signaling, p66Shc signaling in the liver was suppressed and accompanied by lower ROS production and apoptosis [96]. Moreover, a patient cohort with NASH showed significantly higher hepatic protein expression levels of p53, p21 and p66Shc compared to a group with simple steatosis, which again argues for an increase of p53 signaling in NASH that correlates with its severity. Corroborating this, p53 protein levels were elevated in concordance with the extent of liver fibrosis in these patients.

As stated above, the development of fibrosis portends a poor outcome in NASH and can be seen as precursor pathology to cirrhosis and HCC. A major driver of hepatic fibrosis is the activation of quiescent HSCs [73], which turn into proliferating, fibrogenic myofibroblasts [78]. This activation is mediated by chronic liver damage and results in excessive ECM production and fibrotic scarring [97]. Importantly, promotion of senescence of activated HSCs can blunt fibrotic tissue formation, and p53 plays a pivotal role in this pathway. Krizhanovsky et al. [73] found that CCl_4_-induced liver fibrosis was aggravated in p53-deficient mice, a phenotype that was mediated by an attenuation of the senescence program in HSCs.

Another mechanistic link of p53 to liver fibrosis was shown in two different fibrosis mouse models, using atherogenic diet or repetitive thioacetamide injection. Those treatments led to an induction of connective tissue growth factor (CTGF) mRNA. CTGF is a cytokine that is highly upregulated in fibrotic human liver and that correlated with p53 activation [98], suggesting p53 as a possible activator of fibrogenic pathways. Hepatocyte-specific Mdm2 deletion in mice also led to increased p53 protein levels and concomitant CTGF synthesis, hepatocyte apoptosis and spontaneous liver fibrosis. Ablation of p53 completely abolished this fibrotic phenotype. The authors therefore suggest that p53 activation increases hepatocyte apoptosis, which is accompanied by elevated CTGF synthesis, together leading to HSCs activation and liver fibrosis development [98].

In summary, p53-mediated mechanisms described in NASH culminate either in apoptosis of hepatocytes or senescence of HSCs (Figure 1). Several studies suggest an involvement of the JNK/miRNA-34a/Sirt1/p53 pathway, ultimately leading to an onset of apoptosis, which was linked to both intrinsic (mitochondrial ROS formation) and extrinsic (bile acids, death receptor expression) factors. Hence, p53 activation in hepatocytes could promote the onset of NASH by increasing tissue stress and inflammatory response. In contrast, p53-mediated HSCs senescence was shown to have beneficial effects by blunting excess liver fibrosis caused by proliferating HSCs. These opposing findings reflect the complexity of p53-dependent regulation in NASH.

## 5. Multifunctional Roles of p53 in Hepatocellular Carcinoma

HCC mostly occurs in cirrhotic livers, while in approximately 20% of cases, it can occur without preexisting cirrhosis [25,99,100]. The predominance of risk factors leading to HCC development is strongly determined by the geographic region, as well as race and ethnicity [99,101]. In Asia and sub-Saharan Africa, the most common risk factors are still DNA damaging agents, such as aflatoxin B1 or hepatitis B (HBV) or C virus (HCV) infections [99,101]. In Western countries, the rise in HCC incidences in recent years and the projected rise in the coming years can be attributed to altered lifestyle and dietary habits, which favor high caloric nutrition known to cause obesity and type 2 diabetes, as well as to increased alcohol intake known to cause alcoholic liver disease. All of these factors have been suggested to increase the risk of HCC via development of NAFLD and NASH [99,102,103].

To reveal the pathophysiological contribution of p53 in the development of HCC, several mouse models have been developed in the last two decades. It is now well established that p53 impinges on the entire microenvironment of HCC, i.e., affecting hepatocytes, HSCs, immune cells, as well as cancer stem cells (CSCs). All stages of HCC development from initiation to metastasis are affected by p53 [99,104]. Therefore, alterations in p53 signaling pathways are considered as pro-oncogenic by acting on cell proliferation, survival, invasion and immune evasion [99].

Altered expression or mutations of the TP53 gene have been reported with high incidence (found in 12–48% of all HCC) and poor prognostic value in human HCC [99,105,106,107]. Besides deletion and loss-of-function mutations, inactivation of p53 can be achieved via alteration of its upstream regulators such as Mdm2, Mdm4 and wild-type p53-induced phosphatase 1 (Wip1) [108]. Of the known inducers of p53 mutations, the most prominent ones are HBV or HCV and aflatoxin B1, the latter causing the R249S hotspot mutation [109]. Globally, distinct p53 mutation patterns have been observed in HCC patients from different geographical areas [110]. Analysis of p53 mutations in cirrhotic liver samples revealed regional differences of p53 mutations in regenerative (healthy) nodules and in cancerous tissue from the same patient. This confirmed the independent monoclonal origin of distinct regenerative nodules, as well as the susceptibility of highly proliferating liver cells to p53 mutation as a potential link between cirrhosis, liver regeneration and HCC [111].

A few studies demonstrated that p53 deletion can cause HCC development by inducing dedifferentiation of mature hepatocytes, as well as by bi-directional differentiation of liver progenitor cells. A mouse model showing that p53 deletion as a single genetic lesion in liver is sufficient to induce tumor formation was developed by Katz et al. [112]. Conditional p53 deletion, by use of the Cre/loxP system under control of the albumin/α-fetoprotein enhancer/promoter, targets hepatocytes and liver progenitor cells and occurs on embryonic days 10–11. This led to the development of liver carcinomas with a bi-lineal origin, consisting of albumin-positive, hepatocytic and cytokeratin-19-positive cholangiocytic cells in 14–20-month-old mice [112]. Deletion of p53 also dysregulated retinoblastoma checkpoint genes and caused chromosomal aberrations [112]. Furthermore, the bi-lineal tumor differentiation was attributed to the induction of liver progenitor cell differentiation, as well as to dedifferentiation of hepatocytes, which were both mediated by the loss of p53, although the downstream targets of p53 involved in the dedifferentiation process were not revealed [112]. A complex study, using several mouse models to interfere with p53 activity, demonstrated that the origin of the bi-lineal tumor lies in common progenitor cells (oval cells) expressing nestin that are present in adult livers and are activated by chronic liver damage [113]. Nestin expression is restricted by p53 via Sp1/3, thus limiting the cellular plasticity and tumorigenesis of progenitor cells, as well as dedifferentiation of mature hepatocytes in response to oncogenic stimulus [113]. The bi-lineal origin of liver tumors has also been demonstrated in triple transgenic, Trp53/Ink4a/Arf-null mice [114]. Furthermore, p53 was shown to negatively regulate the renewal of hepatic CSCs by inhibiting Nanog, through a mechanism involving mitophagy, a form of autophagy that selectively removes mitochondria [115].

The crosstalk between p53-induced senescence and the immune response is one of the main mechanisms of tumor clearance. In one of the studies exploring the role of p53 in the immune response arising in HCC, embryonic liver progenitor cells transduced with retroviruses expressing H-RasV12 oncogene and p53 shRNA driven by a tet-off promoter were transplanted into adult livers of athymic nude mice [116] or injected subcutaneously into Rag2^−/−^ mice [117]. Mutual H-RasV12 expression and p53 downregulation in the absence of doxycycline resulted in HCC development. When p53 was re-activated by doxycycline administration in developed HCC, a rapid (within 12 days) involution of carcinomas was detected. Interestingly, when p53 was again suppressed, the cancer reappeared [116]. Rather than directly inducing apoptosis, p53 exerted a cytostatic effect by blocking proliferation and induced senescence of cancer cells, rendering them susceptible to recognition by the immune system [116,117]. Cancer removal was effectuated by immune cells, which were recruited by upregulated inflammatory cytokines in senescent cancer cells. Among the recruited cells, macrophages (recruited via colony stimulating factor 1 (Csf1) and monocyte chemoattractant protein 1 (Mcp1)), neutrophils (via Cxcl1) and NK cells (via interleukin-15 (IL-15)) were detected [116,117,118].

Non-hepatocyte tumor suppressive effects of p53 are mediated by its activity in HSCs [118]. In this model, p53 was conditionally inactivated in HSCs, and liver fibrosis was induced by CCl_4_ treatment. Consequently, loss of p53 resulted in increased fibrosis due to increased expansion of activated HSCs and excessive ECM production and, eventually, led to tumor occurrence and liver failure [118]. In line with the hypothesis of tumor-promoting alterations in the local microenvironment, HCC did not directly evolve from p53-depleted HSCs, rather its deletion in HSCs created a niche for malignant transformation of hepatocytes. This was achieved by reduced senescence of HSCs and impaired immune surveillance [118]. Unlike in tissue homeostasis, the signals from the tumor microenvironment, such as TGF-β or IL-4, induce the differentiation of tumor-promoting M2 macrophages [119]. Importantly, p53-expressing HSCs were shown to stimulate the tumor-inhibiting M1 phenotype of macrophages in HCC, while p53-depleted HSCs stimulated differentiation of macrophages into the M2 phenotype [118]. Taken together, the aforementioned results support a model whereby physiological p53 activity in hepatocytes and HSCs facilitates an anti-tumor microenvironment resulting in tumor clearance [116,117,118]. However, it is important to stress that these experiments were performed in immunologically-compromised mice, i.e., athymic nude mice and Rag2^−/−^ mice, in which an appropriate immune response was absent.

HCC usually occurs due to accumulated mutations in the background of liver injury [120]. In this context, animal models mimicking liver injury and/or overexpression of oncogenes in parallel with p53 deletion were developed. Aiming to create a physiological animal model of liver fibrosis-dependent HCC, transgenic mice with cMyc oncogene overexpression and simultaneous p53 knock-down were treated with CCl_4_ [121]. This setting was sufficient to drive the development of HCC [121,122,123]. The described model is valuable in the research needed to investigate the cross-talk of p53 and the underlying mechanisms of HCC development in relation to fibrosis. In another model, hepatic delivery of the potent murine oncogene PyMT (polyoma virus middle T antigen) into liver-specific p53 knock-out mice resulted in the development of invasive carcinomas, which metastasize into the lung [124]. Out of 100 differentially-expressed genes in p53 wild-type compared to p53 knock-out mice, three genes that are known be involved in human metastatic HCC were pointed out: cathepsin E, insulin like growth factor 2 (Igf2) and Igf2 binding protein 2 (Igfbp2) [124]. The cell autonomous role of p53 in the development of invasive HCC was further established by Chen et al. using the same p53 knock-out mouse model [114]. This study revealed that deletion of tumor suppressor Ink4a/Arf in addition to p53 loss accelerates tumorigenesis and metastatic potential. Interestingly, while a cell line derived from the p53-null tumor showed poor migration and invasion capacity in vitro, these cells still efficiently colonized the lungs of immunocompromised nude mice after intravenous injection, confirming their metastatic potential [114].

Transgenic mice expressing the SV40 large T antigen (TAg), which binds to p53 and attenuates its downstream signaling [125], progressively develop HCC and die at 21–26 weeks of age [126,127]. In the livers of SV40 TAg transgenic mice, overexpression of p53 inhibited the development of pre-neoplastic lesions or dysplastic cells, both being morphological alterations that frequently appear in SV40 TAg transgenic mice prior to HCC development [104]. Of note, p53 expression dosage had no effect on the time of tumor onset, and overexpressing p53 in the liver did not show altered liver development and homeostasis, even though some of the p53 target genes were upregulated [104]. Even though the authors suggested that p53-independent, SV40 TAg-affected pathways, such as inhibition of retinoblastoma tumor suppressor (Rb) functions, can be a possible explanation for these divergent results [104], the conclusions derived from this study should be taken with caution since no p53 protein levels were shown to compare the levels between wild-type and overexpressed p53 in mouse livers, and the interplay between SV40 TAg and p53 has not been resolved in detail. Yan et al. investigated HCC development after chronic diethyl nitrosamine (DEN) treatment in p53 wild-type and p53^+/−^ rats [128], as opposed to single injection DEN commonly used to induce HCC in animals. This chronic DEN exposure led to sustained induction of p53 in wild-type livers, while the levels of p53 in the livers of heterozygous rats were, as anticipated, significantly lower, similar to levels observed in non-treated wild-type mice [128]. Somewhat unexpectedly, this led to enhanced hepatocarcinogenesis in p53 wild-type rats, while the size and number of tumors detected in p53^+/−^ animals were significantly lower. The authors argue that sustained activation of p53 after chronic DEN treatment leads to increased hepatocyte apoptosis and subsequent induction of a strong inflammatory response mediated by high-mobility group box 1 (HMGB1) release, which favors tumor development by attracting immune cells or stimulating neighboring cells to release pro-inflammatory cytokines [128]. These studies underline the importance of precise regulation of p53 expression levels in the development of HCC and liver injury in general: in addition to other DEN-initiated effects, sustained high expression of p53 may lead to strong inflammation and liver damage, which in turn induces compensatory hepatocyte proliferation rendering the tissue more susceptible to tumor development [128]. On the other hand, moderate activation of p53, as seen in p53 heterozygous rats, maintains low inflammation levels, thus creating a non-tumorigenic microenvironment. Importantly, increased levels of wild-type p53 have been detected in fibrotic livers and in dysplastic liver nodules in humans, particularly in patients with viral hepatitis [129,130]. In addition, p53 hyperactivation after hepatocyte-specific Mdm2 deletion induced spontaneous liver fibrosis in mice [98]. A recent study showed that coordinated modulation of p53, Sirt1 and Pgc1α activity during the course of HCC development is regulated by the C/ebpβ-Hdac1 complex. The authors proposed a model whereby the level of p53 protein increased in early stages, while being reduced in later stages of HCC development initiated by single DEN injection in mice [131].

**Figure 2:**
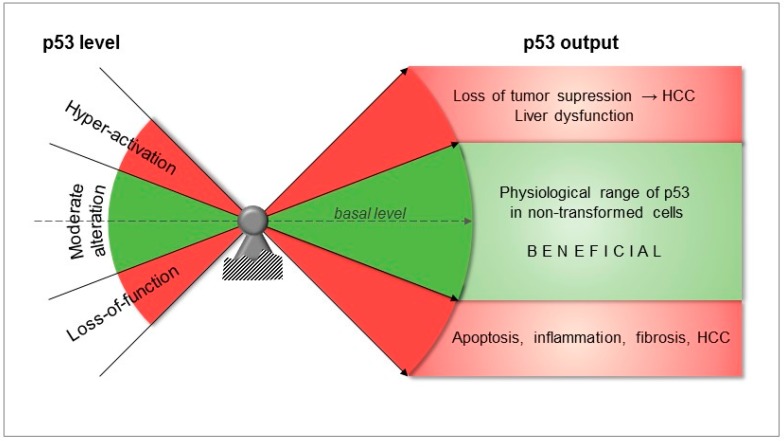
The importance of p53 levels in liver physiology and pathology. Refer to the text for further details. Abbreviations: HCC, hepatocellular carcinoma.

Taken together, moderate changes in the level of p53 maintain normal homeostasis of liver tissue, while a significant decrease, deletion or, on the other hand, chronic hyper-activation of p53 can lead to cancer initiation (Figure 1 and Figure 2).

## 6. p53 in the Regulation of Liver Regeneration

Liver regeneration occurs at a very slow pace in healthy liver, while injury induced by a variety of insults results in release from quiescence and dynamic turnover. The remarkable regenerative capacity of the liver can be accredited to the fact that different hepatocyte sub-populations within the liver possess self-renewal capacity, although the distinction between populations is still debated [132]. Unipotent Axin-2-positive hepatocytes participate in homeostatic renewal, while bipotent periportal hepatocytes participate in liver regeneration induced by chronic damage [132,133]. In response to acute damage, quiescent hepatocytes can be activated to proliferate, while hepatic progenitor cells (HPCs) are able to divide if hepatocyte proliferation is blocked by replicative senescence (reviewed in [133]). The regenerative process is regulated, among many other players, by p53 via modulation of cell cycle check points, cellular plasticity, ploidy and senescence.

β-naphthoflavone (βNF)-inducible Mdm2 deletion in adult mouse livers led to p53 hyperactivation in more than 98% of hepatocytes followed by rapid hepatocyte death, senescence and severe liver injury [134]. In this model, regeneration was elicited by HPCs positive for pan-cytokeratin (panCK), epithelial cell adhesion molecule (EpCAM) and CD24 observed throughout the liver parenchyma, which was in contrast to their predominant localization proximal to the biliary tree in wild-type mice. HPCs did not show upregulated p53, confirming that they were not affected by the transgenic system in response to the βNF induction. To overcome rapid development of jaundice, Mdm2 haploinsufficient mice were used as a long-term model of liver injury, and complete liver regeneration originating from HPCs was observed after six months in these mice. Furthermore, ex vivo cultivated HPCs isolated from Mdm2 haploinsufficient mice were able to regenerate severely injured liver after being introduced by splenic injection into Mdm2 knock-out mice [134]. Interestingly, no tumors were detected in any of the mice after liver regeneration, even though increased rates of proliferation might render the liver prone to cancer development as discussed in the previous paragraph. Another study, using Wip1-null mice, demonstrated that the lack of Wip1, which inhibits both p53-p21 and mTORC1-S6K pathways, results in sustained activation of mTORC1, which counteracts the anti-proliferative effect of the p53-p21 axis, thus leading to liver regeneration after partial hepatectomy (PH) [135]. In agreement with previous data, a two-stage model for liver regeneration after acetaminophen-induced hepatotoxicity was proposed by Fan et al. In the first stage, p53 induction early after injury led to apoptosis and necrosis. In the second stage, p53 was downregulated to permit cell proliferation, resulting in liver regeneration [136]. The two-stage model could partly be explained by the notion that during the course of liver regeneration, p53 binding affinity to its target genes, and therefore their regulation, can vary. Global profiling of hepatic gene expression in regenerating versus quiescent liver has demonstrated that p53/TA-p73 binding to the forkhead factor Foxo3 promoter in quiescent, healthy hepatocytes led to increased Foxo3 expression and further to inhibited cell proliferation [137]. Following PH, when hepatocytes enter the S phase of the cell cycle, the binding affinity of p53/TA-p73 on the Foxo3 promoter was reduced, resulting in a boost of hepatocyte proliferation. The p53/TA-p73 binding affinity to Foxo3 and their regulatory functions were restored after liver regeneration was complete [137].

Seemingly in contrast to the previously-discussed studies showing that p53 hyperactivation leads to liver regeneration, a regeneration-restricting role of p53 has also been proposed in connection to jun proto-oncogene (c-jun) regulation [138]. As demonstrated after PH in double transgenic mice where both c-jun and p53 were knocked out, p53 loss led to improved liver regeneration and increased animal survival in comparison to mice with only c-jun deficiency. The authors argue that c-jun acts to keep p53 activity at basal levels during liver regeneration, therefore preventing the G1/S cell cycle block. Hence, knock-out of c-jun evokes p53 hyper-activation that can inhibit cell proliferation and hence liver regeneration. This study suggested a complex regulatory network consisting of c-jun, p53, p38 and p21, as well as p53-independent mechanisms implicated in liver regeneration and provided evidence of p53 activity regulation during liver regeneration [138]. Furthermore, consistent with previous results, mice in which only p53 was knocked out showed higher viability after PH compared to c-jun knock-out mice, as well as a higher proliferation rate of hepatocytes.

Cellular fate determination towards either liver regeneration or HCC development after injury is a sensitive process, and p53 seems to be a determinant in the fine-tuning of the balance between these two fates (reviewed in [139]). Depending on the cause and level of injury, HPCs can drive liver regeneration, but can also give rise to cancer cells. When liver injury was induced by a choline-deficient, ethionine-supplemented diet in p53-null mice, lack of p53 activated HPCs that were capable of giving rise to HCC in athymic nude mice [140]. Increased expression of Aurora-A, a serine-threonine kinase important for mitotic progression, can lead to polyploidy and aneuploidy that can cause carcinogenic transformation of cells. In one study investigating this scenario, a protective role of p53 was demonstrated: a p53-dependent cell cycle checkpoint was activated after PH to cause cell cycle arrest and prevent transformation of hepatocytes into regenerating cells in livers of Aurora transgenic mice [141]. Furthermore, moderate inhibition of p53 expression by antisense oligonucleotides induced liver regeneration after PH in mice, due to a release in the G1/S checkpoint. This supports the hypothesis that p53 protects the regenerating liver from over-proliferation, a state that can evoke HCC development [142].

Hepatocytes in the mammalian liver are prone to polyploidy and aneuploidy, with approximately 40% of polyploid cells in human liver and 90% in murine liver [143]. During liver regeneration, multiple cell divisions increase the need for ploidy check points. Kurinna et al. compared mitotic fidelity and ploidy resolution in quiescent and regenerating livers of wild-type p53 and p53 knock-out mice [144]. Deficiency of p53 resulted in decreased numbers of diploid hepatocytes and increased percentage of octaploid cells. After PH, the polyploidy was further increased in p53 knock-out mice. In addition, the dynamics of liver regeneration, i.e., periods of increased proliferation, was different in p53 wild-type and null mice, with p53 deficiency causing more frequent mitotic errors as evidenced by abnormal mitotic figures and lagging chromosomes. In this study, five genes were identified as p53 targets during liver regeneration: Aurka, Foxm1, Lats2, Plk2 and Plk4 [144]. One possible mechanism of p53′s regulation of ploidy in liver cancer development could be its ability to trigger cell cycle check points in response to DNA damage by inhibiting cyclin B1 and CDK1 [145]. When p53 is knocked out, the cells re-enter the cell cycle, and accumulating DNA damage furthers polyploidy [146]. Importantly, the detection of genes regulating the ploidy of cancer cells can lead to therapeutic interventions, such as inhibitors targeting Plk4 [147].

Liver injury also affects p53 in HSCs. After chemical damage through CCl_4_ treatment, p53 levels increase to induce senescence of HSCs, thus allowing clearance of the scar tissue by immune cells. In the absence of p53, HSCs are activated, stimulated to produce ECM, and proliferate, but are not efficiently removed by the immune system, thus resulting in the formation of large fibrotic scars [73].

Based on current literature, increased activity of p53 upon liver injury can be viewed as a balancing factor between regeneration and malignant transformation: it regulates cell cycle check points to protect from transformation and induces apoptosis of damaged cells, while activating progenitor cells for compensatory liver repopulation (Figure 1).

## 7. Fine-tuning of Intracellular p53 Protein Levels

The current evidence points to a pivotal role of p53 in liver disease, whereby a controlled degree of activation seems beneficial in many stages of the pathogenesis, while complete loss of function, but also hyper-activation appear to be detrimental and could promote liver disease and HCC (Figure 2). The cellular p53 levels are meticulously controlled by an intricate, multilayered feedback control system which ensures that p53 protein abundance is kept within certain margins and prevents errant activation. Moderate activation of p53 is an essential cellular stress response basically enabling repair mechanisms to operate while the cell cycle is halted [17]. This creates a tumor-inhibiting microenvironment that can result in hepatic cancer cell clearance [116,117,118,128,148]. On the other hand, both complete absence as well as hyper-activation of p53 activity in the liver generates tumor-supporting microenvironments, thus providing a stimulus for cancer development [112,113,114,148,149] and metastasis [114,124]. In addition, increased levels of p53 have been observed in fibrotic livers and in dysplastic liver nodules in humans [129,130]. The two extremes, absence and hyper-activation of p53, are most likely to occur in patients due to loss-of-function mutations in the p53 gene or due to alterations in p53 upstream regulators, respectively. The most prominent mechanism by which p53 is kept within physiological margins is the negative feedback from Mdm2. Others include interaction with other endogenous inhibitors like Mdm4 or Wip1 [150]. Mdm2 is both a p53 target and an inhibitor. Persistent chronic or extreme stress may disrupt this negative feedback loop and could promote p53 hyper-activation, thereby instigating apoptosis both by transactivating apoptotic genes [151] and by cytoplasmic interaction with mitochondrial BclXL and Bcl2 proteins [19]. This concept is evident in a mouse model of induced, hepatocyte-specific Mdm2 gene deletion. In this model, Mdm2 deletion is followed by a massive increase of p53 protein with concomitant gross hepatocyte death and senescence. The severely injured liver is then repopulated by progenitor cells with a functional p53/Mdm2 axis [134]. Underlining the importance of this regulation in humans, the disruption of Mdm2 activity by a homozygous mutation and the consequent high levels of p53 have been suggested to explain the premature aging phenotype in a patient with segmental progeroid syndrome [152].

Overexpression studies in mice showed that increased p53 improves glucose and insulin tolerance [58,59]. In this context, metabolic pathways could well be part of the system controlling p53 levels. In particular, nutrient-sensitive pathways, such as AMPK, mTOR, PI3K/Akt or SIRT1, have all been shown to interact with p53 in various cell types [22]. In liver cells, these pathways could function as sensors relaying the abundance of certain nutrients and/or nutrient classes to p53 signaling, which then coordinates downstream programs that support survival under nutrient stress. One recent example from our laboratories showed that AMPK, known to phosphorylate p53 at serine residues [153], signals to p53, thereby regulating its stabilization under starvation conditions in hepatocytes [38]. Thus, nutrient-dependent pathways could aid the fine-tuning of cellular p53 levels in physiological scenarios in non-cancerous cells and tissues, as opposed to a rather coarse regulation by Mdm2 and similar regulators of p53 in the context of tumor suppression. A potential mechanism to achieve a high degree of fine-tuning is through post-translational modifications (PTMs) such as phosphorylation or acetylation of p53 [55].

In support of this notion, transgenic mice bearing a serine-to-alanine mutation at Ser18, a major p53 phosphorylation site that is crucial for its stabilization, develop normally and do not show a cancer phenotype at an age when severe hepatic insulin resistance becomes apparent [56,58,154]. Another example of N-terminal mutation eliciting a metabolic phenotype without tumorigenesis is evident in humanized P72R mice that show a differential propensity to liver dysfunction determined by the presence of a proline or an arginine at amino acid position 72 (see above, [155]). In general, polymorphisms of p53 could be conceived as another level of fine regulation of intracellular p53 levels, especially if an exchange of a single amino acid does not result in a complete loss- or gain-of-function event, but rather in alterations of downstream activity. This is in line with the fact that most cancer hotspot mutations in the p53 gene are found in the DNA-binding domain, which contains very few known PTM sites. In fact, data from yeast transactivation assays show that these hotspot mutations are strongly associated with a loss of target gene transactivation [20]. In contrast, mutations in the N- and C-termini that harbor the major bulk of PTM sites only marginally affect transactivation efficiency [20], elicit comparably small phenotypic effects in transgenic mouse models [156] and, thus, are likely to affect only fine-tuning of p53 levels.

In addition to modulation by PTMs, a nuanced regulation of p53 signaling can be achieved by its interaction with micro-RNAs [157]. miRNAs are known to regulate fine-tuning of target mRNAs and to establish robustness in biological processes by forming feed-back loops with their targets [158]. Moderate downregulation of p53 signaling by direct binding to regions in the 3′UTR of p53 mRNA has been reported for a number of miRNAs [157]. In liver carcinoma-derived HepG2 cells, miR-1228 and miR-24 have been shown to target the p53 3′UTR. In both cases, metastatic potential was affected by the miRNA-mediated downregulation of p53 [159,160]. Via indirect mechanisms, miRNAs can also function to activate p53. For example, the liver-specific miR-122* has been shown to target Mdm2 and with that to increase p53 levels moderately, leading to decreased tumorigenesis in xenotransplanted HepG2 cells with ectopic miR-122* overexpression [161]. This is consistent with a significant reduction of miR-122*, which is inversely correlated with Mdm2 expression, in human malignant HCC samples [161]. Similarly, such networks consisting of p53, miRNAs and their targets have been identified in different liver diseases and often constitute feedback loops [44,71,94]. However, substantially more work is needed to elucidate the plethora of physiological interactions of certain miRNAs and p53 and their implications on the output of the p53 signaling pathway in the development of liver diseases.

Collectively, based on the herein reviewed literature, we propose that fine-tuning of intracellular p53 activity, coordinated by PTMs, polymorphisms, as well as miRNAs, could ensure a nuanced regulation of downstream pathways as opposed to tumor suppression (e.g., through apoptosis) mediated by more pronounced (de-)regulation (Figure 2). Thus, activating hepatic p53 levels within a physiological frame might be a worthwhile therapeutic target in a variety of liver pathologies.
